# Heterogeneous Functional Dielectric Patterns for Charge‐Carrier Modulation in Ultraflexible Organic Integrated Circuits

**DOI:** 10.1002/adma.202104446

**Published:** 2021-09-21

**Authors:** Koki Taguchi, Takafumi Uemura, Naoko Namba, Andreas Petritz, Teppei Araki, Masahiro Sugiyama, Barbara Stadlober, Tsuyoshi Sekitani

**Affiliations:** ^1^ SANKEN (The Institute of Scientific and Industrial Research) Osaka University 8‐1, Mihogaoka Ibaraki Osaka 567‐0047 Japan; ^2^ Advanced Photonics and Biosensing Open Innovation Laboratory National Institute of Advanced Industrial Science and Technology (AIST) 2‐1 Yamadaoka Suita Osaka 565‐0871 Japan; ^3^ Graduate School of Engineering Osaka University 2‐1 Yamadaoka Suita Osaka 565‐0871 Japan; ^4^ JOANNEUM RESEARCH Forschungsgesellschaft mbH MATERIALS‐Institute for Surface Technologies and Photonics Franz‐Pichler‐Straße 30 Weiz 8160 Austria

**Keywords:** charge‐carrier modulation, flexible electronics, functional dielectric patterns, integrated circuits, organic transistors, polymer dielectrics, threshold voltage control

## Abstract

Flexible electronics have gained considerable attention for application in wearable devices. Organic transistors are potential candidates to develop flexible integrated circuits (ICs). A primary technique for maximizing their reliability, gain, and operation speed is the modulation of charge‐carrier behavior in the respective transistors fabricated on the same substrate. In this work, heterogeneous functional dielectric patterns (HFDP) of ultrathin polymer gate dielectrics of poly((±)endo,exo‐bicyclo[2.2.1]hept‐ene‐2,3‐dicarboxylic acid, diphenylester) (PNDPE) are introduced. The HFDP that are obtained via the photo‐Fries rearrangement by ultraviolet radiation in the homogeneous PNDPE provide a functional area for charge‐carrier modulation. This leads to programmable threshold voltage control over a wide range (−1.5 to +0.2 V) in the transistors with a high patterning resolution, at 2 V operational voltage. The transistors also exhibit high operational stability over 140 days and under the bias‐stress duration of 1800 s. With the HFDP, the performance metrics of ICs, for example, the noise margin and gain of the zero‐*V*_GS_ load inverters and the oscillation frequency of ring oscillators are improved to 80%, 1200, and 2.5 kHz, respectively, which are the highest among the previously reported zero‐*V*_GS_‐based organic circuits. The HFDP can be applied to much complex and ultraflexible ICs.

## Graphical abstract

Modulating the behavior of charge carriers in respective transistors on the same substrate is essential in maximizing the performance of organic integrated circuits (ICs). Heterogeneous functional dielectric patterns for charge‐carrier modulation are obtained by UV patterning. Consequently, the threshold voltage is precisely controlled within the 2–4 V operational range. Furthermore, these ultraflexible organic ICs exhibit the highest performance.

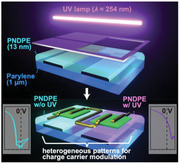

## 1 Introduction

Flexible electronics have gained considerable attention in the development of next‐generation electronic devices for wearable or implantable healthcare monitoring,^[^ ^1^, ^2^, ^3^, ^4^, ^5^, ^6^, ^7^, ^8^ ^]^ and memory or energy storage,^[^ ^9^, ^10^ ^]^ which are accompanied by the exponentially increasing demands of the Internet of Things technology. Flexible devices exhibit multifunctionality and their applicability has been expanded in the recent years owing to the unique properties of organic materials, such as biodegradability^[^ ^11^, ^12^ ^]^ and stretchability.^[^ ^13^, ^14^, ^15^ ^]^ This allows for easier access to useful unobtained information that has not been extracted by conventional silicon technology. Flexible electronic devices often require integrated circuits (ICs) for signal processing; thus, the development of flexible ICs is crucial in the practical applications of flexible electric devices.

Organic thin‐film transistors (OTFTs) are attractive candidates in the development of flexible ICs because they can be fabricated on flexible, and even stretchable substrates, with low‐cost and large‐area fabrication processes.^[^ ^16^, ^17^, ^18^ ^]^ Their individual electric properties have been analyzed and reported over the past few decades, focusing on threshold voltage (*V*_th_),^[^ ^19^, ^20^, ^21^, ^22^, ^23^, ^24^, ^25^, ^26^, ^27^, ^28^ ^]^ mobility,^[^ ^29^, ^30^, ^31^ ^]^ and stability,^[^ ^32^, ^33^ ^]^ which were primarily controlled or improved by modulating the behavior of charge carriers at the interface between the gate dielectrics and semiconductors. Particularly, *V*_th_ is one of the key characteristics which affects the performance of the ICs considering that in the early stages the silicon‐based ICs was produced using only *V*_th_ controlled n‐channel transistors.^[^ ^34^ ^]^ Therefore, the *V*_th_ control using charge‐carrier modulation is essential in OTFTs as well as silicon transistors.

To maximize the performance metrics of the ICs, for example, the reliability, amplification gain, and operation speed, the modulation of the behavior of charge carriers in the respective transistors on the same substrate is desired because it improves the degree of freedom of the circuit design. This can be achieved by providing functional area patterns for charge‐carrier modulation. In the previous studies, the *V*_th_ of the transistors has been controlled by introducing additional gate structures,^[^ ^19^, ^20^ ^]^ the self‐assembled monolayer or oxygen‐plasma treatment,^[^ ^21^, ^22^, ^23^, ^24^, ^25^, ^26^ ^]^ and functional dielectric doping.^[^ ^27^, ^28^ ^]^ However, these techniques are often hindered by the complicated fabrication process or low patterning resolution during the formation of the charge‐carrier modulation field. A simple and high‐resolution patterning process is essential because ICs typically have hundreds of logic gates and transistors.

Photoinduced surface modulation is a promising technique that can be used to obtain functional area patterns for charge‐carrier modulation. The patterned area is obtained by the ultraviolet (UV) irradiation of photoreactive polymers through a shadow mask, which enables *V*_th_ control in the respective transistors on the same substrate. The photoinduced surface modulation method provides a simple patterning process because the patterned area is obtained by transforming the homogeneous functional dielectric structures into heterogeneous structures via photochemical reactions through the UV treatment. Furthermore, this method potentially improves the patterning resolution to be as small as a few hundreds of nanometers, which corresponds to the half‐wavelength of UV radiation. Only a few studies have reported techniques that modulate the charge carriers via photoinduced surface modulation;^[^ ^35^, ^36^ ^]^ moreover, the efficacy of these techniques are hindered by hysteresis characteristics, high operational voltage, and complicated modulation processes.

This paper presents heterogeneous functional dielectric patterns (HFDP) using poly((±)endo,exo‐bicyclo[2.2.1]hept‐ene‐2,3‐dicarboxylic acid, diphenylester) (PNDPE) as the polymer gate dielectrics. PNDPE layers have already been used in previous studies as the gate dielectric in p‐ and n‐channel based OTFTs.^[^ ^16^, ^37^, ^38^ ^]^ However, in these studies, either rigid glass substrates or very thick PNDPE layers as much as a few µm were used; in addition, ICs were not fabricated. This study demonstrates the HFDP for *V*_th_ control in OTFTs on the same substrate for high‐performance ICs with the use of ultrathin PNDPE gate dielectrics. PNDPE transforms its chemical structure from an aromatic ester to *ortho*‐hydroxyketones through the photo‐Fries rearrangement.^[^ ^37^, ^38^, ^39^, ^40^, ^41^, ^42^ ^]^ In PNDPE gate dielectrics, the charge‐carrier modulation is obtained by the heterogeneous molecules of aromatic ester and *ortho*‐hydroxyketones that act as the functional patterns. This results in the programmable *V*_th_ control of the respective transistors on the same substrate. Additionally, hysteresis‐free transistor characteristics are obtained as this process does not use photoinitiators. Furthermore, PNDPE, owing to the catalytic ring‐opening metathesis polymerization, forms dense, uniform, and ultrathin gate dielectrics. As a result, low operational voltage and high transconductance are achieved. The PNDPE‐based transistors are further implemented in ultraflexible ICs to maximize their performance.

In this work, HFDP are obtained with a high resolution of less than 18 µm. The *V*_th_ of the PNDPE‐based transistors is precisely and programmably controlled over a wide range, from −1.5 to +0.2 V at an operational voltage of 2 V, by varying the dose of the UV treatment. This indicates that both the enhancement and depletion transistors are selectively fabricated on the same substrate, which is crucial for tuning the ICs. Furthermore, dense, uniform, and ultrathin PNDPE gate dielectrics of 14 nm supported by a 6 nm thick AlO*_x_* layer are achieved with this process. To demonstrate that the HFDP maximize the performance of ICs, 21 zero‐*V*_GS_ load inverters and a ring oscillator including 48 transistors are fabricated, in which the *V*_th_ of the respective transistors is spatially controlled with the UV treatment. Consequently, noise margin, amplification gain, and propagation delay of the zero‐*V*_GS_ load inverters are improved to 80%, 1200, and 200 µs, respectively. Additionally, the oscillation frequency of the ring oscillator circuit is a maximum of 2.5 kHz, at a supply voltage of 2–4 V. These performances are the highest in the previously reported zero‐*V*_GS_‐based organic circuits. The HFDP demonstrated in this study can be applied to more complex and ultraflexible ICs for signal processing.

## 2 Results and Discussion

**Figure 1:**
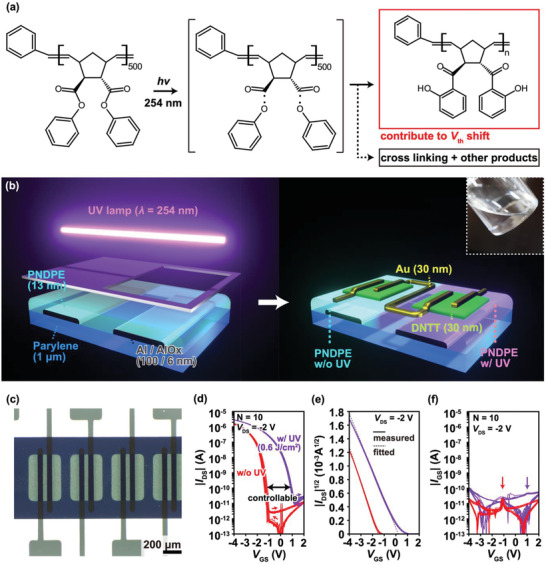
Concept of heterogeneous functional dielectric patterns of poly((±)endo,exo‐bicyclo[2.2.1]hept‐ene‐2,3‐dicarboxylic acid, diphenylester)‐based organic thin‐film transistors (PNDPE‐based OTFTs). Schematics of a) molecular structures of PNDPE via photo‐Fries rearrangement and b) device configuration of OTFTs and the patterning process through UV treatment. Dinaphtho[2,3‐*b*:29,39‐*f*]thieno[3,2‐*b*]thiophene (DNTT) is used as the active semiconducting materials. Inset shows a photograph of PNDPE dissolved in anisole at a concentration of 10 mg mL^−1^. c) Optical image of four PNDPE‐based OTFTs. d) Double sweep transfer curves of the pristine (red lines) and UV‐treated (purple lines) OTFTs at drain−source voltage, *V*_DS_ = −2 V (*N* = 10). e) Square root of drain−source current ($\sqrt{I_{\text{DS}}}$) plots of transfer curves (solid lines) and fitted lines for extrapolation of threshold voltage *V*_th_ in the saturated region method (dashed lines). f) Absolute gate current of the OTFTs (*N* = 10). The arrows indicate the point where the displacement current increases due to the switching of the transistors.

**Figure** **1**a shows the chemical structure and the photoreaction of PNDPE. The preparation of PNDPE was found out to be sustainably scalable, wherein multigram quantities with 73% yield and adequate atom economy can be synthesized at the laboratory level. The synthesis of PNDPE is based on the information presented in a previous report.^[^ ^42^ ^]^ PNDPE contains an aromatic ester group which reacts with UV light through the photo‐Fries rearrangement or radical coupling. The chemical structure of 20% of the total amount of PNDPE is rearranged into *ortho*‐hydroxyketones and other products such as phenol, while the remaining 80% undergoes radical coupling for cross‐linking.^[^ ^42^ ^]^ These *ortho*‐hydroxyketones and phenol contribute to the *V*_th_ shift of the transistors.^[^ ^37^ ^]^ The PNDPE‐based bottom‐gate, and top‐contact OTFTs are fabricated as shown in the right part of Figure 1b. A 1 µm thick ultraflexible parylene substrate is used in this study. The processes are described in detail in the experimental section and Figure S1, Supporting Information. PNDPE is dissolved in anisole as shown in the inset of Figure 1b. Since anisole, being a low‐viscosity solvent, is suitable for printing processes, for example, ink‐jet printing, a sustainable fabrication process is expected owing to the minimum wastage of the material. In addition, its solution is visibly transparent because its absorption occurs in the UV range.^[^ ^41^, ^42^ ^]^ This transparency is potentially useful in the application of imperceptible electronics.^[^ ^43^ ^]^ In this study, the PNDPE solution is spin‐coated to form ultrathin gate dielectrics, in which the thickness is 13.6 ± 0.5 nm (the number of sample: *N* = 9) on an aluminum gate electrode, which is anodized to form an AlO*_x_* layer (thickness: 6 nm). The thickness of each layer was determined through the capacitance measurement (more details are mentioned in Section 4). The PNDPE gate dielectrics are then treated with UV lamp under N_2_ gas, as shown in the left part of Figure 1b. As shown in Figure S2, Supporting Information, the capacitance slightly increases with the UV irradiation (0.60 J cm^−2^) because the dielectric constant of PNDPE changes with the chemical structure.^[^ ^38^ ^]^ Subsequently, dinaphtho[2,3‐*b*:29,39‐*f*]thieno[3,2‐*b*]thiophene (DNTT) and the gold source and drain electrodes are thermally evaporated through a shadow mask. An optical image of the fabricated transistors is shown in Figure 1c, in which the channel length (*L*) and the channel width (*W*) of transistors are *L* = 50 µm and *W* = 500 µm, respectively.

The electric characteristics of the pristine and UV‐treated transistors are measured to validate the charge‐carrier modulation, that is, the *V*_th_ shift, through UV treatment. The electric measurements are performed under ambient and dark conditions, unless otherwise noted. The transfer curves of the ten transistors in Figure 1d indicate that the *V*_th_ of the transistors is shifted under the UV treatment with negligible hysteresis and low characteristic variation. Additionally, a high on‐off ratio of over 10^5^ is obtained independent of the UV treatment. The output curves in Figure S3, Supporting Information, also indicate negligible hysteresis, independent of the UV treatment. An ohmic contact is obtained with both the pristine and UV‐treated transistors. In this study, *V*_th_ is calculated using the extrapolation method in the saturated region from the square root drain current ($\sqrt{I_{\text{DS}}}$) plots (dashed lines in Figure 1e). Here, the calculated *V*_th_ does not exhibit a significant error, considering that the reliability factor is ≈80% (the calculation of the reliability factor has been mentioned in Section 4). As shown in Figure 1f, the gate current (*I*_GS_) is maintained in the order of pA, with and without UV illumination. Since *I*_GS_ is attributed to the displacement current and leakage current, the contributions of these currents to *I*_GS_ were investigated as shown in Figure S4, Supporting Information. The contribution of the displacement current can be estimated by increasing the measurement delay time. We estimated the displacement current at *V*_GS_ = −2 V, and it is ±2 pA in the pristine and ±30 pA in the UV‐treated transistors. Furthermore, since the leakage current significantly increases at *V*_GS_ = +2 and −4 V, the leakage current is estimated at most ≈15 pA in the pristine and ≈300 pA in the UV‐treated transistor. Here we note that the estimated leakage currents include displacement current, but its contribution is up to 15% from the above estimations. Also, although the increase in *I*_GS_ implies that the UV illumination slightly degrades the dielectric properties, it does not significantly affect the transistor operation. The steep displacement current, which is the indication of turn‐on voltage *V*_on_, is observed at *V*_GS_ = +1.0 and −1.1 V, with and without UV treatment, respectively. Additionally, *V*_th_ shifts from negative to positive, as indicated by the dashed lines in Figure 1e. Therefore, both the depletion and enhancement transistors are obtained by using charge‐carrier modulation, originating from the photo‐Fries rearrangement in the PNDPE gate dielectrics. In the practical IC fabrication, the preparation of both types of transistors is desirable on the same substrate, that is, the enhancement transistors is crucial in switching, and the depletion transistors is useful for analog and digital circuits.^[^ ^44^ ^]^

**Figure 2:**
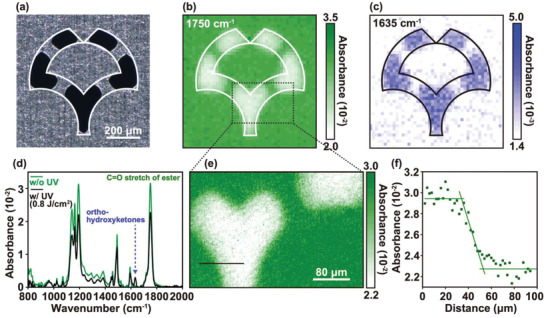
Fourier transform infrared spectroscopy (FT‐IR) measurements of poly((±)endo,exo‐bicyclo[2.2.1]hept‐ene‐2,3‐dicarboxylic acid, diphenylester) (PNDPE) films to investigate the patterning resolution of photo‐Fries rearrangement. a) An optical image of a fabricated shadow mask in the logo of Osaka University. FT‐IR images constructed at the absorbance peak at b) 1750 cm^−1^ and c) 1635 cm^−1^ respectively. The scale of each image is the same as described in (a). The measured pixel size (focal‐plane array: FPA) is 16 µm. d) FT‐IR spectra of the pristine (green line) and UV‐treated (black line) PNDPE films. e) A FT‐IR image in the area surrounded by the dashed line described in (b). The pixel size (FPA) is 2 µm. f) The line profile of the absorbance along the black solid line as drawn in (e).

One of the main advantages of this technique is that it enables the formation of patterns for charge‐carrier modulation with high patterning resolution. Fourier transform infrared spectroscopy (FT‐IR) imaging is performed to analyze the resolution of the HFDP. **Figure** **2**a shows the optical image of a shadow mask patterned in the form of the logo of Osaka University used for the UV patterning process. Figure 2b,c shows the corresponding FT‐IR images of the PNDPE films that are constructed at the absorbance at 1750 and 1630 cm^−1^, which originate from the vibrations of the typical C=O stretch of the ester units and the *ortho*‐hydroxyketones, respectively.^[^ ^41^, ^42^ ^]^ The decrease in the ester units and the formation of *ortho*‐hydroxyketones under UV treatment (0.8 J cm^−2^) are observed in the FT‐IR images. The contrast in the intensity between the pristine and UV‐treated areas is distinct, and the signal intensities of both the areas are uniform, which qualitatively indicates that each area is stoichiometrically uniform. The decrease in the ester units and the formation of the *ortho*‐hydroxyketones are also verified by the FT‐IR spectra, as shown in Figure 2d, where ≈30% of the aromatic ester is transformed into *ortho*‐hydroxyketones or has experienced radical coupling considering the decreased absorbance peak at 1750 cm^−1^ from 0.032 to 0.022. Only a fraction of the PNDPE films react with the UV radiation at the dose of 0.8 J cm^−2^ because this peak almost disappears owing to the nearly complete photo‐Fries rearrangement or the coupling.^[^ ^41^, ^42^ ^]^ The absorption peak at 1197 cm^−1^ is also typical of ester units, and the peaks at 1491 and 1592 cm^−1^ are typical of aliphatic groups, which are also decreased by rearrangement or radical coupling. The patterning resolution is analyzed using the expanded FT‐IR image, as described in Figure 2e. The expanded images are constructed at 1750 cm^−1^ for better contrast. Figure 2f shows the line profile of the absorbance along the black line in Figure 2e. The fitted line is drawn from the slope of the intensity change and the patterning resolution is calculated by measuring the distance from the maximum to minimum intensity. Consequently, the patterning resolution is estimated to be 18 µm. Since the spatial resolution of the measurement method is limited to a value as low as ≈6 µm, the resolution of the HFDP can be assumed to be less than 18 µm. It should be noted that the resolution is limited, in particular, by the thickness (20 µm) of the metal shadow mask. The patterning resolution can be further improved by using collimated UV light, a photomask, or by introducing electron beam lithography. The maximum patterning resolution is expected to be in the sub‐micrometer or even nanometer regime.^[^ ^37^ ^]^

**Figure 3:**
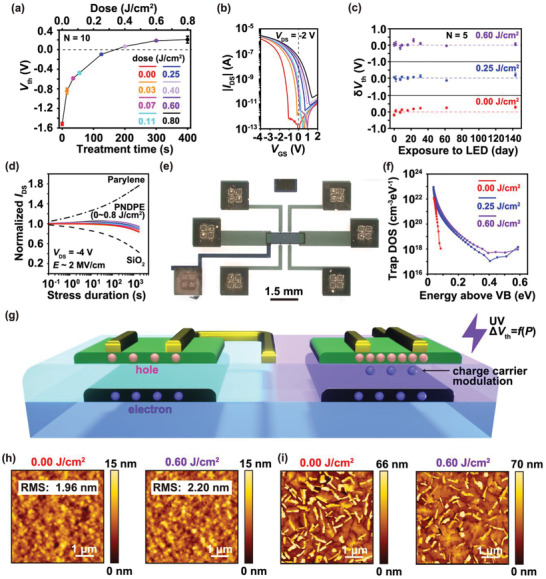
Electric and morphological properties of poly((±)endo,exo‐bicyclo[2.2.1]hept‐ene‐2,3‐dicarboxylic acid, diphenylester)‐based organic thin‐film transistors (PNDPE‐based OTFTs). a) *V*_th_ of the OTFTs as the function of treatment time (UV‐illumination dose) (*V*_DS_ = −2 V). b) Transfer curves of OTFTs at respective doses that correspond to the plots in (a). c) Unintended *V*_th_ change (*δV*_th_) of the pristine and UV‐treated OTFTs under the exposure by a white LED for 143 days (*N* = 5). The error bars show the standard deviation of the measured *V*_th_. d) Normalized *I*_DS_ of the PNDPE‐ (solid lines), SiO_2_‐ (dashed lines), and parylene‐based (dashed‐dotted lines) OTFTs under the stress duration for 1800 s. Each color corresponds to the doses plotted in (a). The gate voltage was applied in the saturation region (*V*_DS_ = −4 V). e) Optical image of OTFTs for four‐probe method. Two of the four additional probes as well as the source and drain electrodes were used for temperature‐dependent measurement. f) Calculated trap density of state (DOS) of OTFTs as the function of energy above valence band (VB). g) Schematic illustration of charge‐carrier modulation by UV illumination. h,i) Atomic force microscopy (AFM) images of PNDPE gate dielectrics with and without UV treatment (h) and dinaphtho[2,3‐*b*:29,39‐*f*]thieno[3,2‐*b*]thiophene (DNTT) crystals on the respective PNDPE films (i).

Another advantage is that the *V*_th_ is programmably controlled by varying the UV dose during the fabrication process. The transfer curves of 10 transistors are measured with different doses and the *V*_th_ is then calculated from the $\sqrt{I_{\text{DS}}}$ plots. **Figure** **3**a shows the dependence of *V*_th_ on the treatment time (dose), and Figure 3b shows the typical transfer curves for the respective doses. The reliability factors are more than 75% for all the transistors at different doses, as shown in Figure S5, Supporting Information. *V*_th_ is precisely and programmably controlled over a wide range, from −1.5 to +0.2 V at *V*_DS_ = −2 V with a low standard deviation within the range of 10–70 mV. The low variation of the pristine and UV‐treated transistors is also confirmed from the histogram of *V*_th_ as shown in Figure S6, Supporting Information. The *V*_th_ control over the wide voltage range further implies that both enhancement and depletion transistors with low‐voltage operation can be fabricated using charge‐carrier modulation via the photo‐Fries rearrangement with the PNDPE gate dielectrics. Here, it can be noted that the *V*_th_ shift is saturated at a dose of 0.8 J cm^−2^, while the decrease in the C=O bond is only 30% at the same dose, as shown in Figures 2d and 3a. Since the carrier transport is more affected by the interface than the bulk of the gate dielectrics, this suggests that the photo‐Fries rearrangement of the surface of PNDPE gate dielectrics is saturated at this dose, and the untreated PNDPE is present in the bulk of the gate dielectrics.

The stable operation of ICs under low‐voltage conditions is the most crucial characteristic for practical IC applications. Returning the *V*_th_ to its original value during long‐term usage is not desirable for practical applications. Since PNDPE reacts with UV light even after the fabrication, *V*_th_ can possibly shift upon exposure to white light, which is used on a daily basis. The transistors exposed to a white light‐emitting diode (LED) were stored over a period of 143 days and the unintended *V*_th_ change (*δV*_th_) was measured to determine the effect of the exposure of the transistors to the white LED. Figure 3c presents the result of the LED exposure test obtained by plotting the relation between *δV*_th_ and the term under exposure to the white LED. The spectrum of the LED is shown in Figure S7, Supporting Information. *δV*_th_ = 0, as indicated by the dashed lines in Figure 3c, is defined as the average *V*_th_ of the first 10 days because it fluctuates slightly under the different measurement conditions, for example, room temperature and humidity. The *δV*_th_ values after 143 days were 300, 200, and 60 mV at 0, 0.25, and 0.60 J cm^−2^, respectively. The UV‐treated transistors do not change the *V*_th_ even under LED illumination, although a slight increase in the *V*_th_ is observed in the pristine transistors, which can be attributed to their reaction with the UV contained in the LED. The shelf‐life stability of the transistors, that is, the ones stored under dark conditions, is also measured as shown in Figure S8, Supporting Information, which indicates the stability of the *V*_th_ of the transistors, both with and without UV treatment over 143 days, although there seemed to be a slight *V*_th_ change for the samples with a UV‐dose of 0.6 J cm^−2^. Therefore, semipermanent *V*_th_ control is achieved by using the PNDPE‐based transistors. This is attributed to the irreversible charge‐carrier modulation via the photo‐Fries rearrangement, which is completely different from memory devices such as the floating gate structures which temporally shift *V*_th_ through charge injections in the additional gate structures.^[^ ^45^ ^]^

The bias‐stress stability of the pristine and UV‐treated transistors is also analyzed. Figure 3d shows the correlation between the stress duration and *I*_DS_, which is normalized at the initial measured time. Transistors with parylene (thickness: 25 nm) and SiO_2_ (thickness: 300 nm) gate dielectrics are also measured for comparison. The applied gate electric field is comparable with all gate dielectrics (PNDPE: 2.1 MV cm^−1^, Parylene: 1.9 MV cm^−1^, SiO_2_: 2.0 MV cm^−1^) to eliminate the effect of the thickness difference. High stability of the normalized *I*_DS_ = 90% is observed after 1800 s in the PNDPE‐based transistors independent of the dose, while normalized *I*_DS_ = 50% and 180% are observed in case of SiO_2_‐ and parylene‐based transistors, respectively.

Further details of the bias‐stress stability can be discussed from the deep trap density of state (DOS) at the interface. The increase in the deep trap DOS deteriorates the bias‐stress stability by decreasing the normalized *I*_DS_ because once the holes are deeply trapped at the donor states, they are not easily released and do not contribute to the transport. Bare SiO_2_‐based transistors have been widely demonstrated to exhibit deteriorated bias‐stress stability, which is also observed in this experiment. In the case of parylene‐based transistors, the bias‐stress stability is deteriorated by increasing the normalized *I*_DS_, which can be attributed to the gradual increase in the acceptor‐like traps at the interface under a continuous bias.^[^ ^46^, ^47^ ^]^ Since the UV‐treated PNDPE‐based transistors slightly increase the normalized *I*_DS_, it is assumed that the UV‐treated PNDPE gate dielectrics initially generate acceptor‐like traps under the bias. Then the effect of donor‐like traps appears to be predominant under the long bias.

In this way, the characteristics of the transistor can be controlled by charge‐carrier modulation, which includes the effect of trapping. Thus, the trap DOS is analyzed on the basis of temperature‐dependent characteristics of the transistors measured by the four‐probe method. Figure 3e shows the optical image of a transistor for four‐probe measurements, and Figure S9, Supporting Information, shows the resulting temperature‐dependent transfer curves. The Arrhenius plots are obtained from the transfer curves, as shown in Figure S10, Supporting Information. Figure 3f shows the trap DOS versus energy above the valence band (VB) calculated from the temperature‐dependent transfer curves. The trap DOS in the deep region (>0.15 eV) increases with UV treatment. The increase in the trap DOS with UV treatment, accompanied by a positive *V*_th_ shift, indicates the generation of acceptor‐like traps.^[^ ^47^, ^48^, ^49^, ^50^ ^]^ The calculated *V*_th_ shift (Δ*V*_th_) from the trap DOS is +1.7 V, which is consistent with the measured *V*_th_ shift, as shown in Figure 3a (more details mentioned in Discussion S1, Supporting Information), indicating that the charge‐carrier modulation in this study originates from the controlling acceptor‐like traps at the interface under UV treatment. In other words, the UV illumination of PNDPE gate dielectrics produces the acceptor‐like traps for the generation of additional hole carriers in the organic semiconductors, as shown in Figure 3g. A high on‐off ratio is obtained since the charge carriers are modulated only in the vicinity of the interface, which is completely different from the bulk doping of the organic semiconductors.

The ultrathin PNDPE gate dielectrics obtained in this study originate from the dense and uniform film formation. The atomic force microscopy (AFM) images of the PNDPE gate dielectrics are shown in Figure 3h and Figure S11a, Supporting Information. There are no major defects or pinholes in spite of the solution process, which is attributed to its preferable polydispersity index of ≈1.^[^ ^41^ ^]^ PNDPE is synthesized with a single degree of polymerization owing to the ring‐opening metathesis polymerization, and its films do not require other kinds of polymers such as photoinitiators to produce the HFDP. Therefore, ultrathin gate dielectrics of 14 nm are achieved without any major defects or pinholes with the support of a 6 nm thick AlO*_x_* layer. The root mean square (RMS) thickness of 1.96 nm in the pristine and 2.20 nm in the UV‐treated (0.6 J cm^−2^) PNDPE films are attributed to the underlying AlO*_x_* layer, whereby its RMS is 4.2 nm, as shown in Figure S12, Supporting Information. The leveling of the PNDPE film by the spin‐coat decreases the surface roughness to ≈2 nm. The RMS of the intrinsic PNDPE gate dielectric is less than 1 nm.^[^ ^37^ ^]^ The slight increase in the RMS upon UV treatment can be attributed to the residual oxygen in the N_2_ gas condition, which results in degradation of the charge‐carrier mobility, as shown in Figure S13, Supporting Information. The crystal size of DNTT is also analyzed by the AFM measurements, as shown in Figure 3i and Figure S11b, Supporting Information. The crystal size does not significantly change under the UV treatment. This is attributed to the nearly constant total surface energy and RMS of PNDPE gate dielectrics due to UV illumination. Figure S14, Supporting Information, shows the contact angle measurement of the pristine and UV‐treated PNDPE gate dielectrics. The surface energy was calculated from the contact angle using the method of Owens and Wendt, and the calculated results are shown in Table S1, Supporting Information. The total surface energy did not depend on the UV‐dose, and the polar component increased by UV illumination, the tendency of which is consistent with our previous study.^[^ ^37^ ^]^ Since the total surface energy is a dominant factor for crystal growth,^[^ ^51^ ^]^ the surface properties of PNDPE with and without UV illumination do not change the crystal growth of DNTT. In addition, the surface roughness changes the crystal growth of the organic semiconductors,^[^ ^52^, ^53^ ^]^ wherein a study showed that the grain size of DNTT changed along with the RMS from 0.9 nm to 1.8 and 2.7 nm;^[^ ^53^ ^]^ however, the RMS measured in this study is only from 1.96 to 2.20 nm. Therefore, we infer that the UV illumination does not significantly change the crystal growth of DNTT, and that the increase in the trap DOS is irrelevant to the morphological properties of the DNTT crystals.

**Figure 4:**
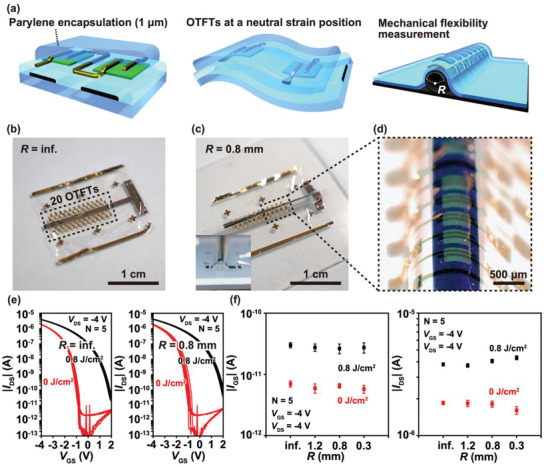
Mechanical flexibility measurement of organic thin‐film transistors (OTFTs). a) Schematic illustration of the measurement method. The bending radius, *R*, varies from the underlying cylinders as shown in the right illustration. b,c) Photographs of the devices including 20 OTFTs on a flat surface (b) and a bent surface (c) along a cylinder with radius 0.8 mm. The inset is the side view of the bent device. d) Optical image of OTFTs bent along with the 0.8 mm radius cylinder. e) Transfer curves of the pristine and UV‐treated OTFTs at the bending radius of infinity and 0.8 mm respectively (*N* = 5). f) |*I*_GS_| and |*I*_DS_| plots as the function of *R* (*N* = 5). The error bars show the standard deviation of the measured characteristics.

Since the PNDPE gate dielectrics and the parylene substrate are ultraflexible, the transistors exhibit high mechanical flexibility. The transfer curves of the PNDPE‐based transistors are measured when the devices are bent with different bending radii. The transistors are encapsulated by 1 µm thick parylene to be placed in the neutral strain position, as shown in **Figure** **4**a, to suppress the strain‐induced changes in the transistor characteristics.^[^ ^54^ ^]^ The bending test is performed by placing the transistors on the cylinders to measure the transfer curves during the bending process. Figure 4b,c present the photograph of a device including twenty transistors on a flat surface and on a bent surface along a cylinder of radius 0.8 mm. The transistors bent by the underlying cylinder are shown in the inset of Figure 4c and the optical image in Figure 4d. Figure 4e depicts the transfer curves of the pristine and UV‐treated transistors when the bending radius is infinity (on a flat surface) and 0.8 mm, respectively. 5 of the 20 transistors are measured during the bending test, in which it is observed that the transfer curves do not vary significantly between the two radii. To further analyze the mechanical flexibility, |*I*_GS_| and |*I*_DS_| are measured by varying the bending radius up to 0.3 mm, which is summarized in Figure 4f. The values of |*I*_GS_| and |*I*_DS_| are almost identical for all the bending radii, where |*I*_GS_| is maintained lower than 6 and 40 pA in the pristine and UV‐treated transistors at a bending radius of 0.3 mm. The change in value of |*I*_DS_| at the bending radius from infinity to 0.3 mm is only ≈10%. Therefore, high mechanical ultraflexibility is achieved by both the pristine and UV‐treated transistors. This is partially attributed to the amorphous film formation of the AlO*_x_* layer as well as the ultrathin AlO*_x_* and PNDPE gate dielectrics. In our previous paper, in which the AlO*_x_* layer was fabricated by the same anodic oxidation method as that in this study, we reported that the dielectric property of the AlO*_x_* layer was maintained even at a bending radius of up to 5 µm.^[^ ^55^ ^]^

**Figure 5:**
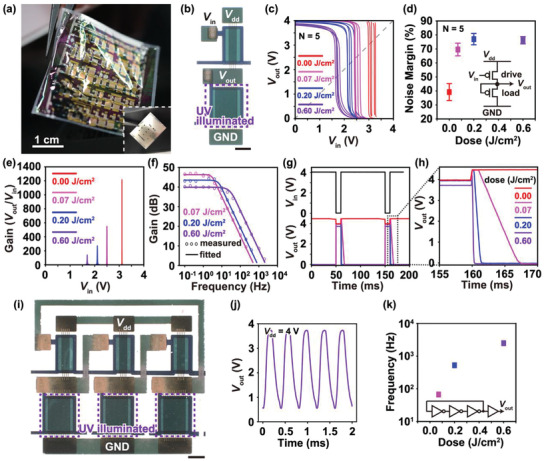
Performances of ultraflexible integrated circuits (ICs). a) Photograph of fabricated zero‐*V*_GS_ load‐inverters and ring oscillators in the area of 3 × 4 cm^2^. The inset is a photograph of a shadow mask to make a pattern of UV treatment. b) Optical image of the zero‐*V*_GS_ load inverter. The load transistor is treated with UV radiation through a shadow mask. Scale bar: 500 µm. c) Inverter characteristics at different UV‐illumination doses (*N* = 5). d) Noise margin of the inverters as the function of the dose (*N* = 5). The error bars show the standard deviation of the measured characteristics. The inset is the schematic circuit diagram of the zero‐*V*_GS_ load inverter. e) Amplification gain of the inverters at different doses. f) Measured (dots) and fitted (solid lines) frequency characteristics of the inverters (*V*_dd_ = 4 V). g) Input and output waveforms of the zero‐*V*_GS_ load inverters running at *f* = 10 Hz and the duty ratio of 10%. h) Output waveforms expanded from (g) in the range of 155–170 ms. i) Optical image of the zero‐*V*_GS_‐based ring oscillator. The size of transistors is identical to (b). Scale bar: 500 µm. j) Output waveform of the ring oscillator treated at 0.6 J cm^−2^. k) Oscillation frequencies of the ring oscillators as the function of the illumination dose. Inset is the schematic circuit diagram of the ring oscillator and an external buffer.

To demonstrate the potential of the HFDP, the PNDPE‐based transistors are implemented in ultraflexible ICs. The zero‐*V*_GS_ load inverters and a ring oscillator including 48 transistors are fabricated on the same substrate in an area of 3 × 4 cm^2^, where the *V*_th_ of the respective transistors are spatially controlled by the HFDP, as shown in **Figure** **5**a,b. The zero‐*V*_GS_ load inverters are promising candidates for flexible organic ICs because they exhibit multi‐functionalities such as high amplification gain in analog circuits and reliable operation in logic circuits by only tuning the *V*_th_ of the transistors, and hence have been widely used as the basic building blocks in the ICs.^[^ ^19^, ^56^ ^]^ It is desirable to implement both the depletion and enhancement transistors in the zero‐*V*_GS_ load inverters for reliable and high‐speed operation. In this study, the load transistor is treated with UV radiation to form a depletion transistor, and the drive transistor is pristine to form an enhancement transistor using a shadow mask, as shown in the inset of Figure 5a. Although a larger width:length (*WL*) ratio of transistors (*L* = 14 µm; *W* = 24 mm) was applied in the circuit application for larger transconductance, nearly the same turn‐on voltage was obtained for different *WL* ratio of transistors. Figure S15, Supporting Information, shows the comparison of transfer characteristics at different WL ratios, for example, *L* = 50 µm; *W* = 500 µm and *L* = 14 µm; *W* = 24 mm. We assumed that the constancy in the turn‐on voltage even for large‐sized transistor is attributed to the uniform PNDPE gate dielectric, at least within the millimeter scale.

The high operational reliability of the zero‐*V*_GS_ load inverters is first demonstrated with the HFDP. Figure 5c shows the five inverter characteristics for four different UV‐illumination doses (0; 0.07; 0.2; and 0.6 J cm^−2^). The trip point is clearly controlled by varying the dose of the UV, in which the respective inverters exhibit a low deviation of less than 90 mV. The measured inverter characteristics are consistent with the simulation results, as shown in Figure S16, Supporting Information. The trip point can be understood by the resistive division between the drive and load transistors. The impedance in the load transistors decreases with the UV treatment because the UV‐treated transistors show depletion characteristics and exhibit higher current when compared to the enhancement transistors at *V*_GS_ = 0. Therefore, the UV treatment of the load transistors shifts the trip point to a lower value. The operational reliability of the respective inverters is estimated using the noise margin, which is defined as “the maximum allowable spurious signal to give correct operation in digital circuits.”^[^ ^57^ ^]^ The noise margin is calculated by using the maximum equal criteria method, as shown in Figure S17, Supporting Information.^[^ ^57^, ^58^ ^]^ Figure 5d shows the noise margin versus the dose of the UV treatment, and the inset shows the schematic circuit diagram of the zero‐*V*_GS_ load inverter. The pristine inverters exhibit a low noise margin of 40%, and the UV treatment improves their noise margin by up to 80%. This value is comparable to that of the previously reported organic complementary inverters.^[^ ^11^ ^]^

Furthermore, the amplification gain and operation speed are programmably tuned in this study. Figure 5e shows the typical static gain of the respective inverters at different doses. The maximum static gain of 1200 is observed for the pristine inverters, although they exhibit ultraslow operation. The UV‐treated inverters also exhibit a high static gain of up to 600. This can be attributed to the small sub‐threshold slope of ≈150 mV and the high transconductance originating from the ultrathin PNDPE gate dielectrics. Here, it is worth noting that the transconductance is further enhanced by implementing the large WL ratio of transistors (*L* = 14 µm; *W* = 24 mm). The frequency characteristics are also analyzed by using the AC measurements, as shown in Figure 5f. Here, an input sine wave is inverted to generate the amplified output signal, as shown in Figure S18, Supporting Information. The UV treatment improved the operation speed, which was a trade‐off relation to the gain. This is theoretically consistent in the common source circuit operation, in which the operation speed and gain are the functions of the impedance and transconductance of the load transistor, respectively. The gain‐bandwidth products (GBWP) in the respective inverters are calculated to evaluate the tradeoffs between the gain and the operation speed. Consequently, the GBWP of up to 2.5 kHz is obtained by the UV treatment of 0.6 J cm^−2^. The GBWP of the other UV‐doses (0.07 and 0.20 J cm^−2^) inverters are 500 and 900 Hz, respectively. The slight mobility degradation owing to the UV treatment is not a significant drawback because the performance of ICs can be compensated by *V*_th_ (or *I*_DS_ at a certain *V*_GS_). In practical applications, high‐gain or high‐speed circuit cells are spatially and arbitrarily implemented in the ICs by utilizing the HFDP.

The operation speed is further analyzed by measuring the signal delay, which is essential in digital circuits. Figure 5g shows the input and output waveforms obtained from the respective inverters. The input signal is a full‐swing pulse wave. In the UV‐treated inverters, the inverted output signals are also observed as full‐swing pulse waves. The expanded output signals in Figure 5h show that the signal propagation delays, that is, the time from the high output state to low output state, are 6.5 ms, 1.4 ms, and 400 µs at 0.07, 0.20, and 0.60 J cm^−2^, respectively. The inverters with more UV‐dose exhibit smaller signal delay, which is consistent with the common source circuit operation. The UV illumination decreases the resistance of load transistors, that is, *I*_DS_ at *V*_GS_ = 0 increases with UV illumination because of the *V*_th_ shift. The overshoot from 4 up to 4.5 V, can be attributed to the discharge of the parasitic capacitance formed between the source and the gate electrodes. The pristine inverters exhibit an almost constant output voltage owing to their ultraslow operation. The output voltage is expected to drop to 0 V after a prolonged measurement.

Finally, this study demonstrates the high‐speed operation of the zero‐*V*_GS_ load inverters by implementing them in ring oscillator circuits. Figure 5i shows an optical image of a 3‐stage ring oscillator circuit. Figure 5j and Figure S19a,b, Supporting Information, show the output waveforms at different doses. The waveforms were extracted via an external inorganic buffer to obtain high input impedance and minimize the parasitic capacitance of the buffer, as shown in the inset in Figure 5k. The oscillation frequencies are 70, 530, and 2.5 kHz, respectively, at UV‐doses of 0.07, 0.20, and 0.60 J cm^−2^, respectively, which is depicted in Figure 5k. This result is supported by the signal delay measurement shown in Figure 5g. Since the ring oscillator circuit consists of three zero‐*V*_GS_ load inverters, in which the delay time is smaller for more UV‐dose, the oscillation frequency is higher for the ring oscillators with more UV‐doses. Since a ring oscillator circuit consisting of the zero‐*V*_GS_ load inverters does not significantly increase the oscillation frequency corresponding to the supply voltage (*V*_dd_) as long as the drive transistor works in the saturation regime, the fabricated ring oscillator is operated at *V*_dd_ = 2 V, without changing its oscillation frequency for all UV‐doses. The waveform of the ring oscillator (UV‐dose: 0.20 J cm^−2^) is shown in Figure S19c, Supporting Information, as an example. The performances of the zero‐*V*_GS_ load inverters and ring oscillators are summarized and compared with those of the previous studies in Table S2, Supporting Information, in which the highest performances obtained in this study are compared with those of the previously reported zero‐*V*_GS_‐based organic circuits.

## 3 Conclusion

This study demonstrated the formation of the HFDP with the use of PNDPE as an ultrathin polymer gate dielectric to modulate the behavior of the charge carriers. The HFDP are obtained with a high‐resolution of less than 18 µm. With the charge‐carrier modulation via the photo‐Fries rearrangement in the PNDPE gate dielectrics, the *V*_th_ of the PNDPE‐based transistors were programmably controlled over a wide range from −1.5 to +0.2 V at an operational voltage of 2 V, indicating that both the enhancement and depletion transistors are arbitrarily fabricated. The modulated *V*_th_ remained unchanged over a period of 140 days during the LED exposure test and under a bias duration of 1800 s. The charge‐carrier modulation was achieved by controlling the acceptor‐like traps. The PNDPE forms dense and uniform gate dielectrics due to the catalytic ring‐opening metathesis polymerization, resulting in ultrathin PNDPE films of a thickness of 14 nm. The transistor also exhibited high mechanical flexibility, in which the characteristics did not significantly vary even at a bending radius of 0.3 mm. The performance metrics of the ICs, for example, the zero‐*V*_GS_ load inverters and the ring oscillator circuits, were maximized with the HFDP. Consequently, the noise margin, gain, and GBWP of the zero‐*V*_GS_ load inverters were improved by up to 80%, 1200, and 2.5 kHz, respectively. Additionally, the oscillation frequency of a ring oscillator circuit was a maximum of 2.5 kHz at a supply voltage of 2–4 V. All of these performance metrics are the highest among the previously reported zero‐*V*_GS_ load‐based organic circuits. The HFDP introduced in this study facilitate the application of more complex, ultraflexible, and wearable ICs for a wide range of applications, including the monitoring of human and structural healthcare.

## 4 Experimental Section

### Materials

Unless otherwise noted, all the commercial reagents and solvents were used as received. Parylene (diX‐SR) was provided by Daisan Kasei (Tokyo, Japan). DNTT was purchased from Nippon Chemical Industrial Co., Ltd (Tokyo, Japan). PNDPE was synthesized according to the details provided in a previous report^[^ ^42^ ^]^ and dissolved in anisole by sonication for several hours at a concentration of 10 mg mL^−1^. The PNDPE solution was stored in the dark for a few days and then filtered through the radius pores of 45 µm.

### Device Fabrication

A 100 nm‐thick Al gate electrode was thermally evaporated through a shadow mask onto a 1 µm thick parylene substrate supported on a fluorinated glass film that was fabricated by the spin‐coating of Cytop. The Al gate electrode was then anodized to form 6 nm thick AlO*_x_* using citric acid (500 mL, 1 mmol) as the weak acid solution and Pt electrode as the cathode. The AlO*_x_* layer was then treated with oxygen plasma at 100 W for 30 s to clean the AlO*_x_* layer. The PNDPE was then spin‐coated at 500 rpm for 5 s and 3000 rpm for the subsequent 20 s to form the gate dielectrics. The PNDPE films were baked at 60 °C for 1.5 h to reduce the residual solvent. A handy‐type Hg lamp (λ = 254 nm, *P* = 0.2 mW cm^−2^ at the sample surface) was used to treat the PNDPE films. Finally, the DNTT and Au source and drain electrodes were evaporated at an evaporation rate of 0.03 and 0.3 nm s^−1^, respectively. The transistors were peeled off for the mechanical flexibility measurement. The transistors were passivated by 1 µm thick parylene films through the chemical vapor deposition in bias‐stress, shelf‐life, and flexibility measurements. The channel length (*L*) and width (*W*) of the transistors were 50 and 500 µm, respectively. The *L* and *W* of the transistors for the four‐probe method were 1.5 mm and 500 µm, respectively, and the distance between the additional two probes was 450 µm. Zero‐*V*_GS_ load inverters were used with the ratio of a driver and a load transistor of 1:3, where *L* and *W* are 14 µm and 8 (24) mm, respectively. The short channel effects were not observed in this study.

### Measurements and Analysis

The capacitance and the thickness of the PNDPE gate dielectrics were evaluated by the capacitance measurements with an LCR meter (E4980A, Keysight Technologies, Inc., Santa Rosa, California, USA). First, the thickness of an AlO*_x_* layer was estimated by means of capacitance measurements, where the relative permittivity of AlO*_x_* was defined as 9. Then the total capacitance of the PNDPE and AlO*_x_* layer was measured and the thickness of the PNDPE layer was extracted as a function of the relative permittivity of PNDPE, which was defined as 2.3 in the pristine. Since the relative permittivity is gradually shifted to 3.0 by the UV illumination, the thickness was estimated from the pristine PNDPE gate dielectrics.^[^ ^38^ ^]^ Given that the thickness of 14 nm is an estimation based on the capacitance measurement, further quantification, for example, transmission electron microscopy, is desirable to clearly evaluate the thickness of the PNDPE gate dielectrics. The transistor characteristics were measured using a semiconductor parameter analyzer (B1500A, Keysight Technologies, Inc., Santa Rosa, CA, USA). The reliability factors were calculated in reference to the previous report.^[^ ^59^ ^]^ The slope of the fitted lines, that is, the transconductance, for calculating the reliability factors, was determined from an average of respective 7 points before and after the point that exhibits maximum mobility. The FT‐IR images were obtained by using a commercial infrared microscope (Hyperion 3000, Bruker Corp., Billerica, MA, USA) under purged N_2_ conditions. The resolution of the focal‐plane array was 2 or 16 µm, and the spectral resolution was 4 cm^−1^. A 25 nm PNDPE film was used as a sample formed on a BaF_2_ substrate coated with a 15 nm thick parylene film. The effect of absorption of the substrate was negated by measuring the FT‐IR spectra of the BaF_2_ and the coated parylene substrate. The illumination tests of the OTFTs with a white LED were carried out under vacuum conditions for ≈7–8 h. The spectra of the LED used can be found in Figure S7, Supporting Information. The temperature measurements were performed using a low‐temperature unit (PEO‐101‐16, Pascal Co., Ltd., Osaka, Japan). The trap DOS was calculated using the fitting equation that was installed with the statistical analysis software (OriginPro, OriginLab Corp., Northampton, MA, USA). The method proposed by Fortunato et al. was used to calculate the trap DOS because it concurs well with the computer simulation model, and more precise consideration was achieved when compared to other calculation methods.^[^ ^60^, ^61^ ^]^ The contact angle measurement was performed by a FACE Measurement and Analytical System (FAMAS, Kyowa Interface Science Co., Ltd, Saitama, Japan). The PNDPE gate dielectrics were formed on the anodized AlO*_x_* layer. The dropped volumes of the water and ethylene glycol were 4 µL.

## Conflict of Interest

The authors declare no conflict of interest.

## Supporting information

Supporting Information

## Data Availability

Research data are not shared.

## References

[1] W. Gao , S. Emaminejad , H. Y. Y. Nyein , S. Challa , K. Chen , A. Peck , H. M. Fahad , H. Ota , H. Shiraki , D. Kiriya , D. H. Lien , G. A. Brooks , R. W. Davis , A. Javey , Nature 2016, 529, 509. 26819044 10.1038/nature16521 PMC4996079

[2] G. Schwartz , B. C. K. Tee , J. Mei , A. L. Appleton , D. H. Kim , H. Wang , Z. Bao , Nat. Commun. 2013, 4, 1859. 23673644 10.1038/ncomms2832

[3] T. A. Ali , J. Groten , J. Clade , D. Collin , P. Schaffner , M. Zirkl , A. M. Coclite , G. Domann , B. Stadlober , ACS Appl. Mater. Interfaces 2020, 12, 38614. 32803962 10.1021/acsami.0c08469

[4] H. Yao , W. Yang , W. Cheng , Y. J. Tan , H. H. See , S. Li , H. P. A. Ali , B. Z. H. Lim , Z. Liu , B. C. K. Tee , Proc. Natl. Acad. Sci. USA 2020, 117, 25352. 32989151 10.1073/pnas.2010989117 PMC7568242

[5] H. Yao , T. Sun , J. S. Chiam , M. Tan , K. Y. Ho , Z. Liu , B. C. K. Tee , Adv. Funct. Mater. 2021, 2008650, 10.1002/adfm.202008650.

[6] S. P. Lacour , G. Courtine , J. Guck , Nat. Rev. Mater. 2016, 1, 16063.

[7] S. Xu , Y. Zhang , L. Jia , K. E. Mathewson , K. I. Jang , J. Kim , H. Fu , X. Huang , P. Chava , R. Wang , S. Bhole , L. Wang , Y. J. Na , Y. Guan , M. Flavin , Z. Han , Y. Huang , J. A. Rogers , Science 2014, 344, 70. 24700852 10.1126/science.1250169

[8] H. Lee , Y. J. Hong , S. Baik , T. Hyeon , D. H. Kim , Adv. Healthcare Mater. 2018, 7, 1701150. 10.1002/adhm.201701150 29334198

[9] Z. Niu , L. Zhang , L. Liu , B. Zhu , H. Dong , X. Chen , Adv. Mater. 2013, 25, 4035. 23716279 10.1002/adma.201301332

[10] K. Qian , R. Y. Tay , M. F. Lin , J. Chen , H. Li , J. Lin , J. Wang , G. Cai , V. C. Nguyen , E. H. T. Teo , T. Chen , P. S. Lee , ACS Nano 2017, 11, 1712. 28112907 10.1021/acsnano.6b07577

[11] A. Petritz , A. Wolfberger , A. Fian , T. Griesser , M. Irimia‐Vladu , B. Stadlober , Adv. Mater. 2015, 27, 7645. 25898801 10.1002/adma.201404627

[12] J. Xu , X. Zhao , Z. Wang , H. Xu , J. Hu , J. Ma , Y. Liu , Small 2019, 15, 1803970. 10.1002/smll.201803970 30500108

[13] M. Kaltenbrunner , G. Kettlgruber , C. Siket , R. Schwödiauer , S. Bauer , Adv. Mater. 2010, 22, 2065. 20354974 10.1002/adma.200904068

[14] A. Hirsch , H. O. Michaud , A. P. Gerratt , S. de Mulatier , S. P. Lacour , Adv. Mater. 2016, 28, 4507. 26923313 10.1002/adma.201506234

[15] M. S. White , M. Kaltenbrunner , E. D. Głowacki , K. Gutnichenko , G. Kettlgruber , I. Graz , S. Aazou , C. Ulbricht , D. A. M. Egbe , M. C. Miron , Z. Major , M. C. Scharber , T. Sekitani , T. Someya , S. Bauer , N. S. Sariciftci , Nat. Photonics 2013, 7, 811.

[16] H. Gold , A. Petritz , E. Karner‐Petritz , A. Tschepp , J. Groten , C. Prietl , G. Scheipl , M. Zirkl , B. Stadlober , Phys. Status Solidi RRL 2019, 13, 1900277.

[17] T. Sekitani , T. Yokota , K. Kuribara , M. Kaltenbrunner , T. Fukushima , Y. Inoue , M. Sekino , T. Isoyama , Y. Abe , H. Onodera , T. Someya , Nat. Commun. 2016, 7, 11425. 27125910 10.1038/ncomms11425 PMC5411732

[18] M. Sugiyama , T. Uemura , M. Kondo , M. Akiyama , N. Namba , S. Yoshimoto , Y. Noda , T. Araki , T. Sekitani , Nat. Electron. 2019, 2, 351.

[19] K. Myny , M. J. Beenhakkers , N. A. J. M. Van Aerle , G. H. Gelinck , J. Genoe , W. Dehaene , P. Heremans , IEEE J. Solid‐State Circuits 2011, 46, 1223.

[20] K. Hizu , T. Sekitani , T. Someya , J. Otsuki , Appl. Phys. Lett. 2007, 90, 093504.

[21] U. Zschieschang , F. Ante , M. Schlörholz , M. Schmidt , K. Kern , H. Klauk , Adv. Mater. 2010, 22, 4489. 20803763 10.1002/adma.201001502

[22] S. Kobayashi , T. Nishikawa , T. Takenobu , S. Mori , T. Shimoda , T. Mitani , H. Shimotani , N. Yoshimoto , S. Ogawa , Y. Iwasa , Nat. Mater. 2004, 3, 317. 15064756 10.1038/nmat1105

[23] U. Zschieschang , V. P. Bader , H. Klauk , Org. Electron. 2017, 49, 179.

[24] K. Fukuda , T. Sekitani , U. Zschieschang , H. Klauk , K. Kuribara , T. Yokota , T. Sugino , K. Asaka , M. Ikeda , H. Kuwabara , T. Yamamoto , K. Takimiya , T. Fukushima , T. Aida , M. Takamiya , T. Sakurai , T. Someya , Adv. Funct. Mater. 2011, 21, 4019.

[25] A. Kitani , Y. Kimura , M. Kitamura , Y. Arakawa , Jpn. J. Appl. Phys. 2016, 55, 03DC03.

[26] R. Shiwaku , Y. Yoshimura , Y. Takeda , K. Fukuda , D. Kumaki , S. Tokito , Appl. Phys. Lett. 2015, 106, 053301.

[27] I. Lashkov , K. Krechan , K. Ortstein , F. Talnack , S. J. Wang , S. C. B. Mannsfeld , H. Kleemann , K. Leo , ACS Appl. Mater. Interfaces 2021, 13, 8664. 33569958 10.1021/acsami.0c22224

[28] H. Wang , P. Wei , Y. Li , J. Han , H. R. Lee , B. D. Naab , N. Liu , C. Wang , E. Adijanto , B. C. K. Tee , S. Morishita , Q. Li , Y. Gao , Y. Cui , Z. Bao , Proc. Natl. Acad. Sci. USA 2014, 111, 4776. 24639537 10.1073/pnas.1320045111 PMC3977307

[29] K. Nakayama , Y. Hirose , J. Soeda , M. Yoshizumi , T. Uemura , M. Uno , W. Li , M. J. Kang , M. Yamagishi , Y. Okada , E. Miyazaki , Y. Nakazawa , A. Nakao , K. Takimiya , J. Takeya , Adv. Mater. 2011, 23, 1626. 21472790 10.1002/adma.201004387

[30] H. Klauk , M. Halik , U. Zschieschang , G. Schmid , W. Radlik , W. Weber , J. Appl. Phys. 2002, 92, 5259.

[31] T. Uemura , Y. Hirose , M. Uno , K. Takimiya , J. Takeya , Appl. Phys. Express 2009, 2, 111501.

[32] U. Zschieschang , F. Ante , T. Yamamoto , K. Takimiya , H. Kuwabara , M. Ikeda , T. Sekitani , T. Someya , K. Kern , H. Klauk , Adv. Mater. 2010, 22, 982. 20217824 10.1002/adma.200902740

[33] U. Kraft , J. E. Anthony , E. Ripaud , M. A. Loth , E. Weber , H. Klauk , Chem. Mater. 2015, 27, 998.

[34] G. S. May , C. J. Spanos , Fundamentals of Semiconductor Manufacturing and Process Control, Wiley‐IEEE, New York 2006.

[35] M. Marchl , M. Edler , A. Haase , A. Fian , G. Trimmel , T. Griesser , B. Stadlober , E. Zojer , Adv. Mater. 2010, 22, 5361. 20931561 10.1002/adma.201002912 PMC3021719

[36] T. T. Dao , H. Sakai , H. T. Nguyen , K. Ohkubo , S. Fukuzumi , H. Murata , ACS Appl. Mater. Interfaces 2016, 8, 18249. 27348479 10.1021/acsami.6b03183

[37] A. Petritz , A. Wolfberger , A. Fian , J. R. Krenn , T. Griesser , B. Stadlober , Org. Electron. 2013, 14, 3070. 24748853 10.1016/j.orgel.2013.07.014 PMC3990428

[38] A. M. Ramil , G. Hernandez‐Sosa , T. Griesser , C. Simbrunner , T. Höfler , G. Trimmel , W. Kern , Q. Shen , C. Teichert , G. Schwabegger , H. Sitter , N. S. Sariciftci , Appl. Phys. A 2012, 107, 985. 10.1007/s00339-012-6853-2 PMC3682804 23785220

[39] G. Hernandez‐Sosa , C. Simbrunner , T. Höfler , A. Moser , O. Werzer , B. Kunert , G. Trimmel , W. Kern , R. Resel , H. Sitter , Org. Electron. 2009, 10, 326.

[40] W. Gu , D. J. Abdallah , R. G. Weiss , J. Photochem. Photobiol., A 2001, 139, 79.

[41] T. Griesser , T. Höfler , S. Temmel , W. Kern , G. Trimmel , Chem. Mater. 2007, 19, 3011.

[42] T. Höfler , T. Grießer , X. Gstrein , G. Trimmel , G. Jakopic , W. Kern , Polymer 2007, 48, 1930.

[43] A. Takemoto , T. Araki , T. Uemura , Y. Noda , S. Yoshimoto , S. Izumi , S. Tsuruta , T. Sekitani , Adv. Intell. Syst. 2020, 2, 2000093.

[44] G. Gelinck , P. Heremans , K. Nomoto , T. D. Anthopoulos , Adv. Mater. 2010, 22, 3778. 20533415 10.1002/adma.200903559

[45] S. J. Kim , J. S. Lee , Nano Lett. 2010, 10, 2884. 20578683 10.1021/nl1009662

[46] K. Fukuda , T. Suzuki , D. Kumaki , S. Tokito , Phys. Status Solidi A 2012, 209, 2073.

[47] S. Scheinert , G. Paasch , M. Schrödner , H. K. Roth , S. Sensfuß , T. Doll , J. Appl. Phys. 2002, 92, 330.

[48] J. A. Merlo , C. D. Frisbie , J. Phys. Chem. B 2004, 108, 19169.

[49] A. R. Völkel , R. A. Street , D. Knipp , Phys. Rev. B 2002, 66, 195336.

[50] S. Scheinert , K. P. Pernstich , B. Batlogg , G. Paasch , J. Appl. Phys. 2007, 102, 104503.

[51] M. Kawamura , Y. Nakahara , M. Ohse , M. Kumei , K. Uno , H. Sakamoto , K. Kimura , I. Tanaka , Appl. Phys. Lett. 2012, 101, 053311.

[52] S. E. Fritz , T. W. Kelley , C. D. Frisbie , J. Phys. Chem. B 2005, 109, 10574. 16852282 10.1021/jp044318f

[53] M. Geiger , R. Acharya , E. Reutter , T. Ferschke , U. Zschieschang , J. Weis , J. Pflaum , H. Klauk , R. T. Weitz , Adv. Mater. Interfaces 2020, 7, 1902145.

[54] T. Sekitani , S. Iba , Y. Kato , Y. Noguchi , T. Someya , T. Sakurai , Appl. Phys. Lett. 2005, 87, 173502.

[55] M. Kaltenbrunner , T. Sekitani , J. Reeder , T. Yokota , K. Kuribara , T. Tokuhara , M. Drack , R. Schwödiauer , I. Graz , S. Bauer‐Gogonea , S. Bauer , T. Someya , Nature 2013, 499, 458. 23887430 10.1038/nature12314

[56] M. Kondo , M. Melzer , D. Karnaushenko , T. Uemura , S. Yoshimoto , M. Akiyama , Y. Noda , T. Araki , O. G. Schmidt , T. Sekitani , Sci. Adv. 2020, 6, eaay6094. 32010789 10.1126/sciadv.aay6094 PMC6976294

[57] S. De Vusser , J. Genoe , P. Heremans , IEEE Trans. Electron Devices 2006, 53, 601.

[58] J. S. Yuan , L. Yang , IEEE Trans. Educ. 2005, 48, 162.

[59] Y. Xu , H. Sun , A. Liu , H. Zhu , B. Li , T. Minari , F. Balestra , G. Ghibaudo , Y. Y. Noh , Adv. Funct. Mater. 2018, 28, 1803907.

[60] G. Fortunato , D. B. Meakin , P. Migliorato , P. G. Le Comber , Philos. Mag. B 1988, 57, 573.

[61] W. L. Kalb , B. Batlogg , Phys. Rev. B 2010, 81, 035327.

