# Supplementary material for: Heterogeneous Functional Dielectric Patterns for Charge‐Carrier Modulation in Ultraflexible Organic Integrated Circuits
Source: Adv Mater. 2021 Sep 21;33(45):2104446. doi: 10.1002/adma.202104446 (PMC11469327; doi:10.1002/adma.202104446)
Supplement: Supplementary file 1 — Supporting Information [file ADMA-33-2104446-s001.pdf]

© 2021 Wiley-VCH GmbH

ADVANCED MATERIALS

Supporting Information

for *Adv. Mater.,* DOI: 10.1002/adma.202104446

Heterogeneous Functional Dielectric Patterns for Charge-Carrier Modulation in Ultraflexible Organic Integrated Circuits

Koki Taguchi, Takafumi Uemura, Naoko Namba, Andreas Petritz, Teppei Araki, Masahiro Sugiyama, Barbara Stadlober, and Tsuyoshi Sekitani**

Supporting Information

Heterogeneous Functional Dielectric Patterns for Charge-Carrier Modulation in Ultraflexible Organic Integrated Circuits

Koki Taguchi, Takafumi Uemura, Naoko Namba, Andreas Petritz, Teppei Araki, Masahiro Sugiyama, Barbara Stadlober, and Tsuyoshi Sekitani**

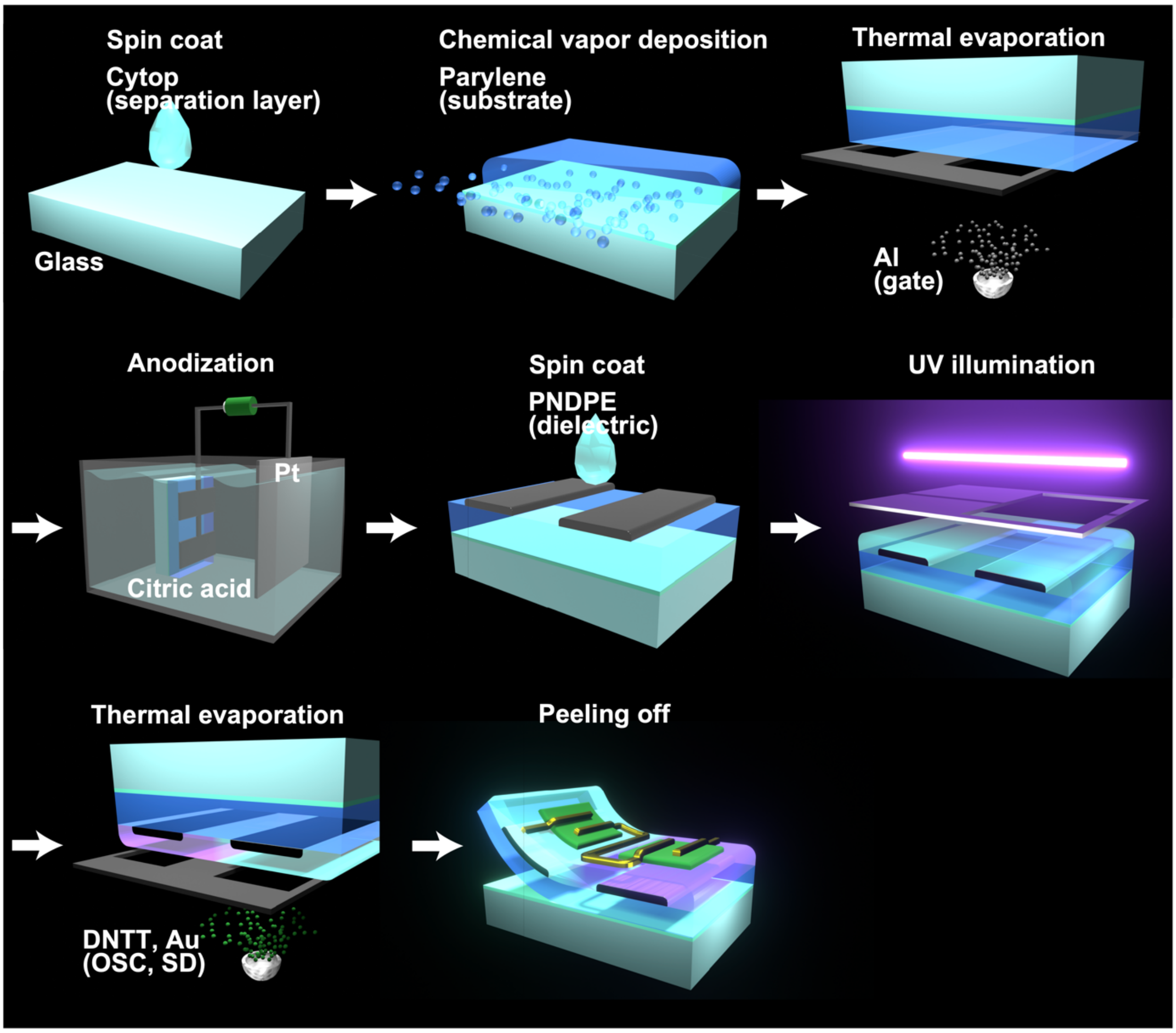

Figure S1. Schematic illustration of the detailed fabrication process of poly((±)endo,exo-bicyclo[2.2.1]hept-ene-2,3-dicarboxylic acid, diphenylester) (PNDPE)-based organic transistors. The textual description is mentioned in the experimental section.

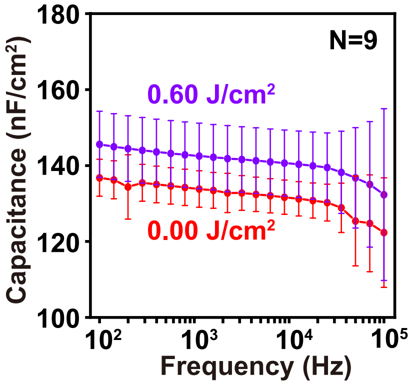

Figure S2. Capacitance versus frequency plots of poly((±)endo,exo-bicyclo[2.2.1]hept-ene-2,3-dicarboxylic acid, diphenylester) (PNDPE), and AlO_x gate dielectrics at different doses (0 and 0.60 J cm^{-2} correspond to the red plots and purple plots, respectively). Error bars show the standard deviation of measured capacitance (N = 9).

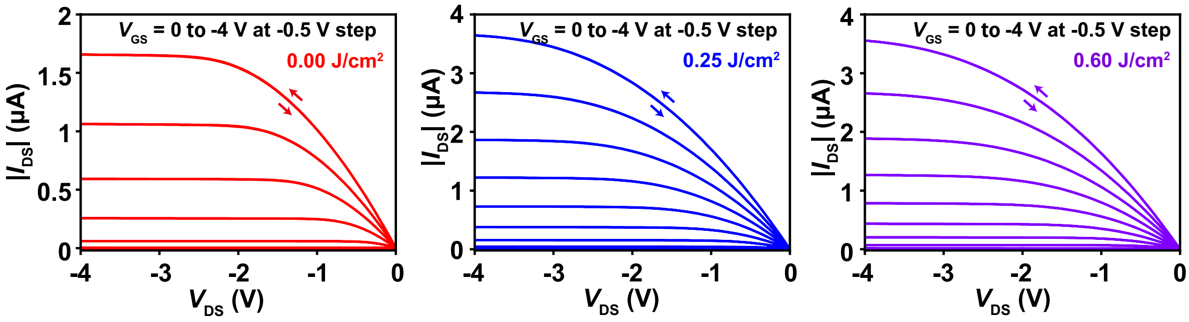

Figure S3. Output curves of poly((±)endo,exo-bicyclo[2.2.1]hept-ene-2,3-dicarboxylic acid, diphenylester) (PNDPE)-based transistors at different doses.

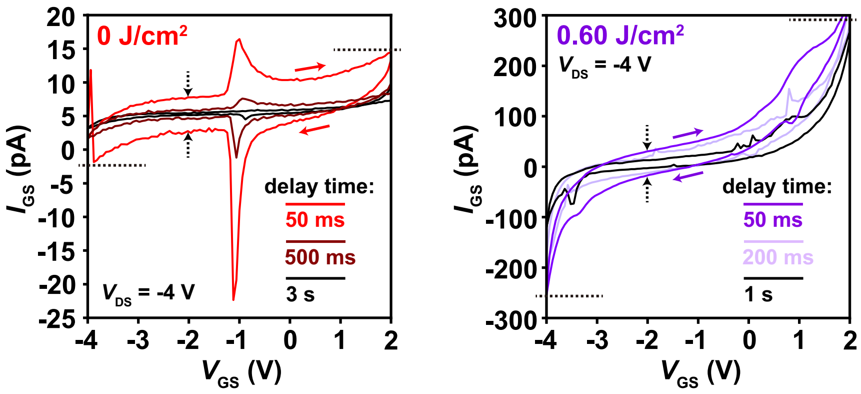

Figure S4. Gate current (I_{GS}) plots of the poly((±)endo,exo-bicyclo[2.2.1]hept-ene-2,3-dicarboxylic acid, diphenylester) (PNDPE)-based transistors with and without UV illumination while varying the delay time of the measurement that is the waiting time after applied V_{GS} is changed. Here, I_{GS} obtained at the delay time of 50 ms is the same condition as Figure 1f, and measurement results with longer delay time are plotted simultaneously. The I_{GS} obtained with the shorter delay time dominantly represents the displacement current; on the other hand, the convergence to the actual leakage current is observed by increasing the delay time. Dashed arrows at V_{GS} = −2 V represent the points of the estimated displacement current, and dashed lines represent the estimated leakage current at V_{GS} = +2 and −4 V.

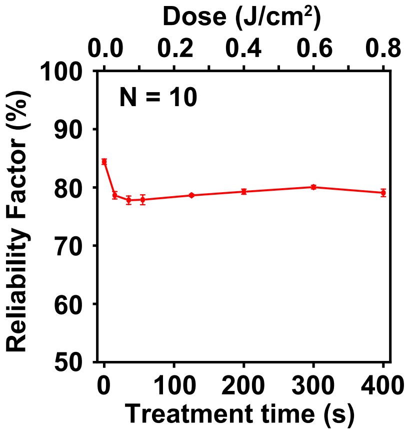

Figure S5. Reliability factor of the fitted lines drawn in square root of drain-source current ($\sqrt{I_{DS}}$) plots corresponding to the treatment time (dose).

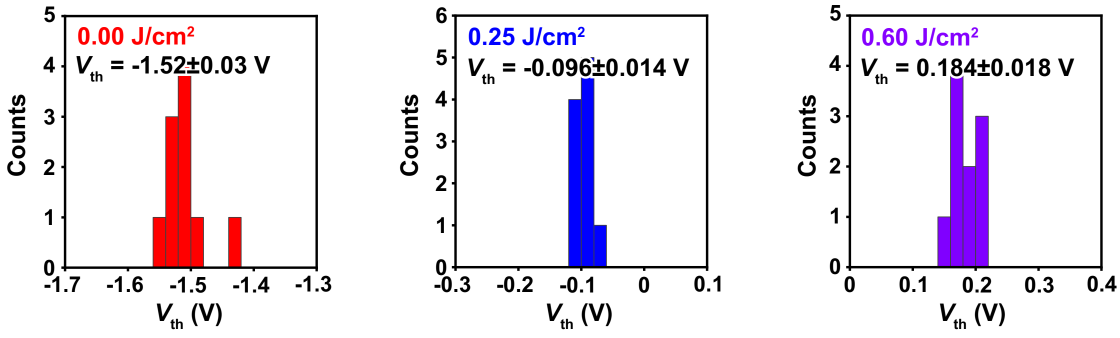

Figure S6. Histogram of threshold voltage (V_{th}) of transistors with respective UV-doses (N = 10).

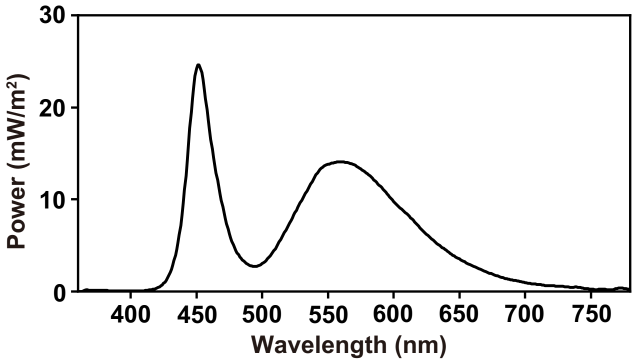

Figure S7. The spectrum of a white light-emitting diode (LED). The illuminance of the LED is 710 lx at the sample surface.

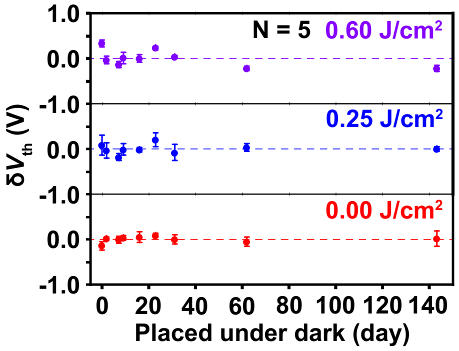

Figure S8. Shelf-life stability in the pristine and UV-treated (0.25 and 0.60 J cm^{-2}) transistors stored in the dark for 143 days.

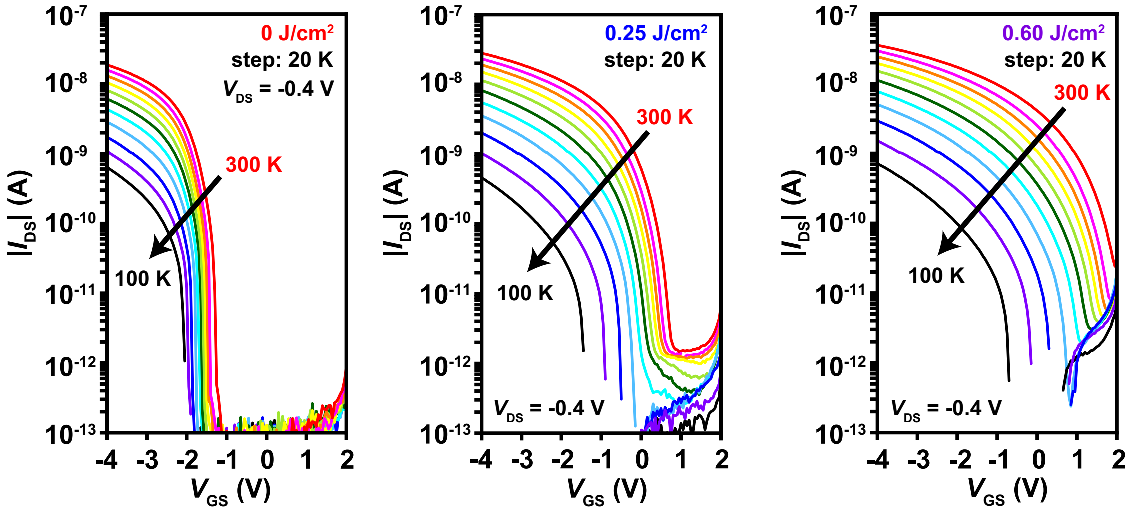

Figure S9. Temperature-dependent transfer curves in respective transistors from 300 K to 100 K in steps of 20 K. Drain-source voltage (V_{DS}) is −0.4 V to measure the carrier transport in the linear region. Transistor characteristics varied from the measured temperature due to the existence of traps in the vicinity of the interface.

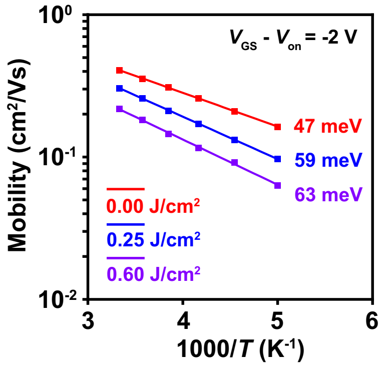

Figure S10. Arrhenius plots with the linear fitted lines calculated from the temperature-dependent transfer curves at different doses. To equally compare the carrier transport, the gate voltage is applied to $V_{GS} - V_{on} = -2$ V (V_{GS}: gate voltage, V_{on}: turn-on voltage of the transistors).

Discussion S1: Calculation of threshold voltage (V_{th}) shift from the trap density of state (DOS)

We compared the measured and calculated V_{th} shifts estimated from the trap DOS to demonstrate that the charge carrier modulation reported in this study is achieved by controlling the acceptor-like traps at the interface. The estimated V_{th} shift (ΔV_{th}) was calculated from the trap DOS as follows:

$$\Delta V_{th} = \Delta N \cdot \frac{q}{C_i} \quad \text{... (S1)}$$

where ΔN is the number of deep traps, q is the elementary charge, and C_i is the unit capacitance of the gate dielectrics. The deep traps (ΔN) increased by the UV treatment significantly affected the V_{th}, while the shallow traps mainly contributed to the carrier transport. The deep traps are defined as the states that are located at energies greater than 0.15 eV above the valence band because the previous studies reported that the traps (less than 0.15 eV) are very close to the valence band and are attributed to the thermal fluctuation originating from the intermolecular transfer integral.[1] In addition, we determined the accumulation layer, which is the area that induces the acceptor-like traps. Since the transfer curves of the UV-treated transistors show an off-state, the induced traps are located only in the vicinity of the interface rather than the bulk of the organic semiconductor.[2] In this study, the accumulation layer was determined to be 5 nm based on a previous study, in which the assumption of only the traps in the 5 nm layer obtained a good fit for the experimental data.[2] Assuming that the generation of donor-like traps is negligible by UV treatment, and that the deep acceptor-like traps in the pristine transistors are negligible, ΔN is calculated by the integral of the trap DOS above 0.15 eV multiplied by the accumulation layer. Consequently, $\Delta N = 1.45 \times 10^{12}$ cm^{-2} was obtained at a dose of 0.60 cm^{-2}. By substituting the calculated ΔN and the unit capacitance of the poly((±)endo,exo-bicyclo[2.2.1]hept-ene-2,3-dicarboxylic acid, diphenylester) (PNDPE) gate dielectric (137 nF cm^{-2}) into Equation. (S1), we obtain the ΔV_{th} of $+1.7$ V. Since the calculated ΔV_{th} is consistent

with the measured ΔV_{th} as shown in Figure 3a, the V_{th} shift observed in this study originates from the acceptor-like traps.

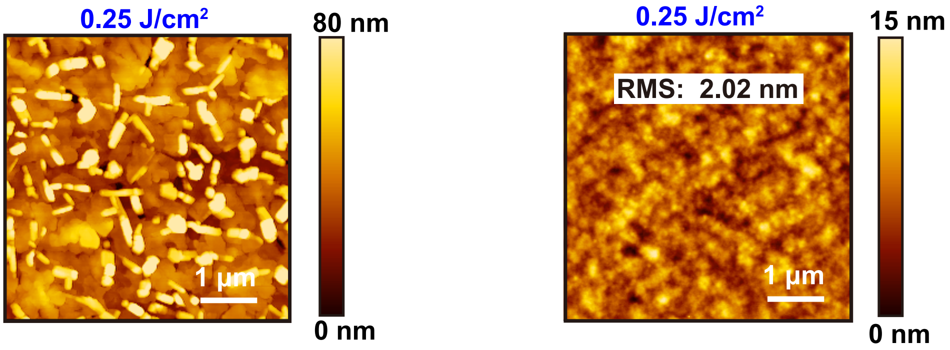

Figure S11. Atomic force microscopy (AFM) images of (a) poly((±)endo,exo-bicyclo[2.2.1]hept-ene-2,3-dicarboxylic acid, diphenylester) (PNDPE) gate dielectric and (b) dinaphtho[2,3-b:29,39-f]thieno[3,2-b]thiophene (DNTT) crystals at the UV-dose of 0.25 J cm^{-2}.

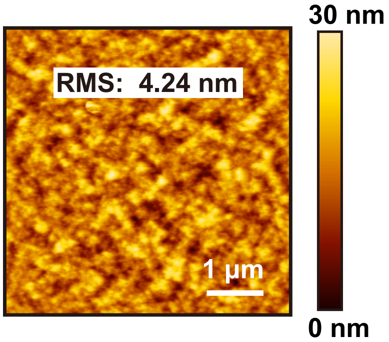

Figure S12. Atomic force microscopy (AFM) image of the anodized AlO_x layer.

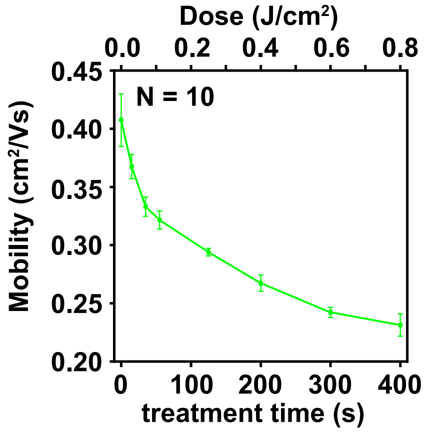

Figure S13. The charge carrier mobility of the transistors corresponding to the treatment time (dose).

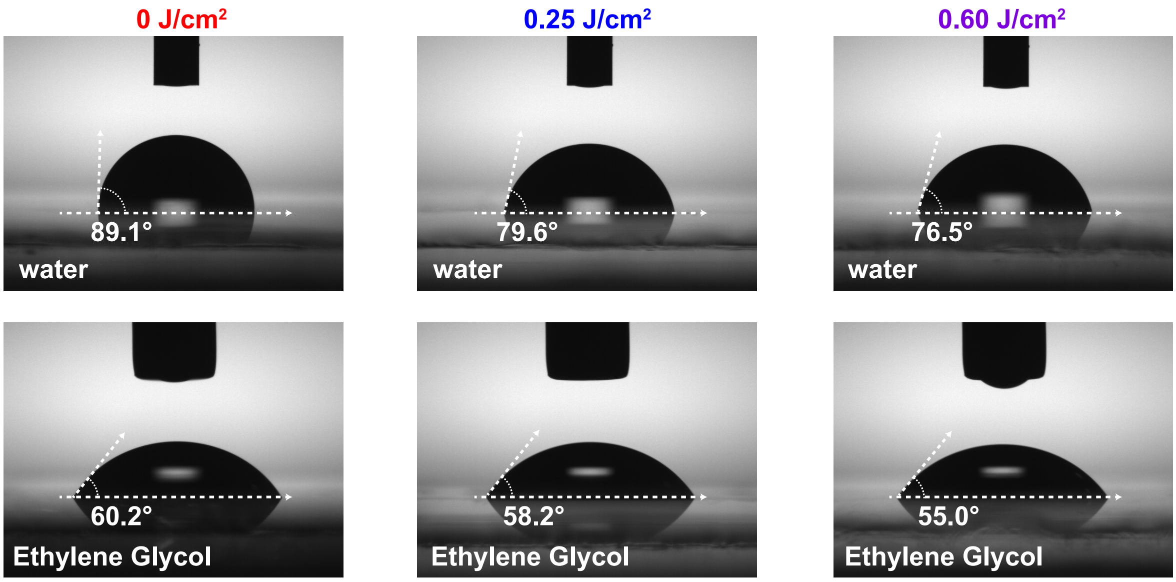

Figure S14. Contact angle of the poly((±)endo,exo-bicyclo[2.2.1]hept-ene-2,3-dicarboxylic acid, diphenylester) (PNDPE) gate dielectrics at different doses.

Table S1. Surface energy of the poly((±)endo,exo-bicyclo[2.2.1]hept-ene-2,3-dicarboxylic acid, diphenylester) (PNDPE) gate dielectrics at different doses.

UV-dose (J cm^{-2})	$γs^p$ (J cm^{-2})	$γs^d$ (J cm^{-2})	γs (J cm^{-2})
**0**	3.25 ± 0.30	26.3 ± 1.0	29.6 ± 0.74
**0.25**	11.2 ± 0.80	16.7 ± 1.2	27.9 ± 0.47
**0.60**	13.1 ± 0.43	16.6 ± 0.67	29.7 ± 0.30

$γs^p$: the polar component in surface energy
$γs^d$: the dispersive component in surface energy
γs: the total surface energy

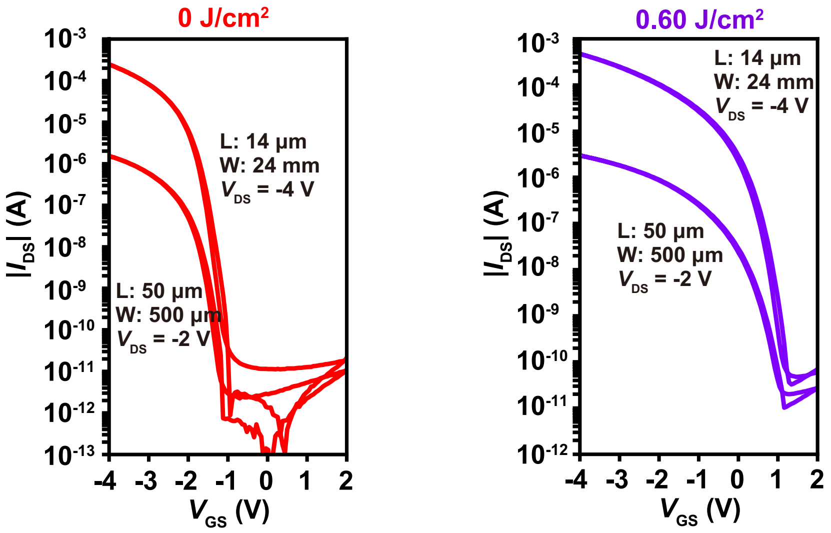

Figure S15. Transfer curves of the pristine and UV-treated transistors at different width:length (WL) ratios.

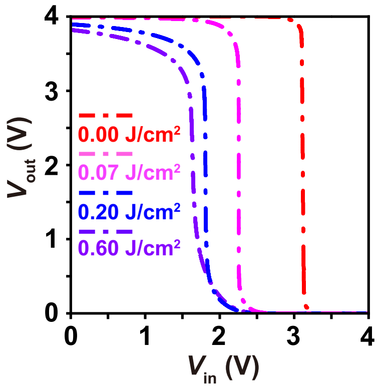

Figure S16. Simulated inverter characteristics of the zero-V_{GS} load inverters at different UV-doses. Simulation was performed by the H-SPICE simulator, in which we created the models of organic transistors from the measured transfer and output curves based on the a-Si transistor model. The gate current (I_{GS}) was also considered in this simulation that can affect the pristine inverter characteristics.

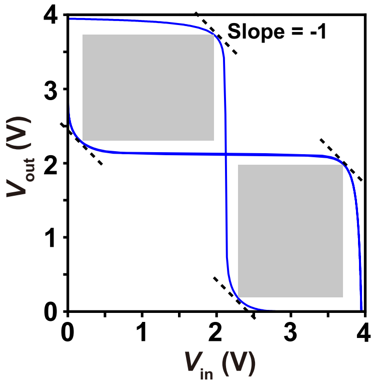

Figure S17. Maximum equal criteria method used to calculate the noise margin. The noise margin is determined by identifying the maximum size of a square obtained from the inverter and mirrored inverter curves. We calculated high and low state noise margins (NM_H and NM_L), respectively. Then the smaller value of either NM_H or NM_L was displayed as noise margin in Figure 5. The inverter curves of the UV-treated zero-V_{GS} load inverter (0.20 J cm^{-2}) are described as the sample.

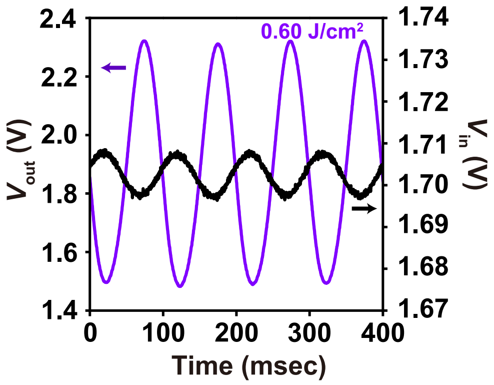

Figure S18. Input and output sine waveforms obtained through the zero-V_{GS} load inverter (0.60 J cm^{-2}). Peak to peak input voltage is 10 mV and the input frequency is 10 Hz.

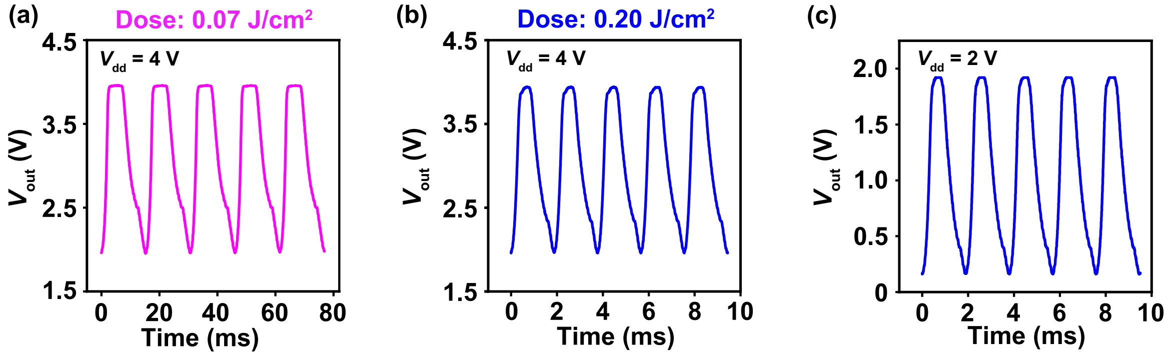

Figure S19. Waveforms of the zero-V_{GS} load-based ring oscillator circuits with the dose of (a) 0.07 J cm^{-2} and (b) 0.20 J cm^{-2}. (c). A waveform of the ring oscillator circuit (treated at 0.20 J cm^{-2}) at 2 V supply voltage.

Table S2. Comparison of performances of inverters between this work and previous works.

	This work	[3]	[4,5]	[6]	[7]	[8]	[9]
**Type**	zero-V_{GS}	zero-V_{GS}	zero-V_{GS}	zero-V_{GS}	CMOS	CMOS	CMOS
**Process for V_{th} control**	Photo-patterning	Photo-reaction	Oxygen-plasma	N/A	Mized SAMs	Photo-reaction	N/A
**Operation voltage**	2-4 V	40 V	20 V	2-4 V	2 V	20 V	2-4 V
**Noise Margin**	80%	N/A	30%	65%	N/A	65%	90%
**Gain**	1200	35	20	250	400	15	500
***Stage Frequency (kHz)**	7.5	N/A	1.5	2.8	N/A	N/A	N/A

V_{GS}: gate voltage of a transistor
CMOS: Complementary metal-oxide-semiconductor
V_{th}: threshold voltage
*Stage Frequency: Oscillation frequency per stage of a ring oscillator

References:

[1] W. L. Kalb, S. Haas, C. Krellner, T. Mathis, B. Batlogg, *Phys. Rev. B* **2010**, *81*, 155315.

[2] A. R. Völkel, R. A. Street, D. Knipp, *Phys. Rev. B* **2002**, *66*, 195336.

[3] M. Marchl, M. Edler, A. Haase, A. Fian, G. Trimmel, T. Griesser, B. Stadlober, E. Zojer, *Adv. Mater.* **2010**, *22*, 5361.

[4] H. Takahashi, M. Kitamura, Y. Hattori, Y. Kimura, *Jpn. J. Appl. Phys.* **2019**, *58*, SBBJ04.

[5] H. Takahashi, Y. Hanafusa, Y. Kimura, M. Kitamura, *Jpn. J. Appl. Phys.* **2018**, *57*, 03EH03.

[6] A. Petritz, M. Krammer, E. Sauter, M. Gärtner, G. Nascimbeni, B. Schrode, A. Fian, H. Gold, A. Cojocaru, E. Karner-Petritz, R. Resel, A. Terfort, E. Zojer, M. Zharnikov, K. Zojer, B. Stadlober, *Adv. Funct. Mater.* **2018**, *28*, 1804462.

[7] U. Zschieschang, F. Ante, M. Schlörholz, M. Schmidt, K. Kern, H. Klauk, *Adv. Mater.* **2010**, *22*, 4489.

[8] T. T. Dao, H. Sakai, H. T. Nguyen, K. Ohkubo, S. Fukuzumi, H. Murata, *ACS Appl. Mater. Interfaces* **2016**, *8*, 18249.

[9] A. Petritz, A. Wolfberger, A. Fian, T. Griesser, M. Irimia-Vladu, B. Stadlober, *Adv. Mater.* **2015**, *27*, 7645.
